# Development, Statistical Optimization, and Characterization of Resveratrol-Containing Solid Lipid Nanoparticles (SLNs) and Determination of the Efficacy in Reducing Neurodegenerative Symptoms Related to Alzheimer's Disease: In Vitro and In Vivo Study

**DOI:** 10.1155/2024/7877265

**Published:** 2024-09-30

**Authors:** Mohammad Amin Khishvand, Ehsan Mehrabani Yeganeh, Mohammad Zarei, Meysam Soleimani, Mojdeh Mohammadi, Reza Mahjub

**Affiliations:** ^1^ Department of Pharmaceutics School of Pharmacy Hamadan University of Medical Sciences, Hamadan, Iran; ^2^ Department of Pharmacology and Toxicology School of Pharmacy Hamadan University of Medical Sciences, Hamadan, Iran; ^3^ Department of Physiology School of Medicine Hamadan University of Medical Sciences, Hamadan, Iran; ^4^ Department of Pharmaceutical Biotechnology School of Pharmacy Hamadan University of Medical Sciences, Hamadan, Iran

**Keywords:** lipid peroxidase (LPO), Morris water maze (MWM), neurodegeneration, oxidative stress, reduced glutathione (GSH), resveratrol, solid lipid nanoparticles (SLNs)

## Abstract

Resveratrol (RSV), as a natural polyphenol exhibiting antioxidative properties, is studied in the treatment of neurodegenerative diseases. However, RSV has low oral bioavailability. In this study and in order to overcome the issue, RSV was encapsulated into the solid lipid nanoparticles (SLNs). In this study, RSV-loaded solid lipid nanoparticles (RSV-SLNs) were prepared by the solvent emulsification-evaporation technique, and their physicochemical properties were optimized using Box–Behnken response surface methodology. The morphology of the particles was evaluated using scanning electron microscopy (SEM) and transmission electron microscopy (TEM). The neuroprotective effects of the nanoparticles were investigated in animal models using the Morris water maze (MWM). Then after, the rats were sacrificed, their brains were collected, and the extent of lipid peroxidase (LPO) as well as the level of reduced glutathione (GSH) were determined in the hippocampus section samples. Finally, the collected brain tissues were histologically studied. The particle size, polydispersity index (PDI), zeta potential, entrapment efficiency (EE%), and drug loading (DL%) of the optimized nanoparticles were 104.5 ± 12.3 nm, 0.322 ± 0.11, −3.1 ± 0.15 mV, 72.9 ± 5.31% and 14.6 ± 0.53%, respectively. The microscopic images revealed spherically shaped and nonaggregated nanoparticles. The *in vivo* studies demonstrated higher efficiency of RSV-SLN in the reduction of escape latency time and improvement in the time spent in the target quadrant compared to free RSV. Moreover, it was demonstrated that RSV-SLN posed a higher potency in the reduction of LPO as well as elevation of the GSH levels in the brain samples. The histological studies revealed a decline in neural degeneration and an improvement in the CA1 pyramidal cell morphology. The obtained data revealed that RSV-SLNs caused more reduction in Alzheimer-related symptoms rather than free RSV.

## 1. Introduction

Alzheimer's disease (AD) is a progressive neurodegenerative disorder and is considered as the most common form of dementia in elderly people. The gradual intercellular condensing of the protein segment amyloid-beta (A*β*), as well as the intracellular accumulation of twisted fibers of the tau proteins, are the key features of the pathogenesis of the disease [1]. Scopolamine (SCO), due to its antagonistic activity on muscarinic acetylcholine (ACh) receptors, is widely studied for the induction of AD in laboratory animal models [2]. Moreover, mitochondrial dysfunction and induction of oxidative stress can be considered as the neurotoxicological mechanisms of SCO-induced AD [3]. In experimental studies, it has been demonstrated that chronic administration of SCO can lead to spatial and memory dysfunction, as well as hippocampal neurodegeneration in rats [4, 5]. It has been shown that the administration of SCO leads to an increase in malondialdehyde (MDA) levels as well as a reduction in reduced glutathione (GSH) levels in animals, indicating the occurrence of oxidative stress [6–8].

Although modulation of neurotransmitters (i.e., ACh and glutamate) is considered as a strategy for the treatment of the disease, several studies have been conducted to attenuate cognitive and memory deficits by reducing oxidative stress in AD animal models. Therefore, the application of natural products exerting antioxidative and anti-inflammatory properties is highly recommended for the treatment of the disease [8].

Natural polyphenols are classified into four main groups including flavonoids, phenolic acids, stilbenes, and lignans [9]. Resveratrol (RSV), a stilbene compound, is commonly found in dietary sources such as grapes, blackberries, peanuts, and red wine [10]. Due to its effects on various molecular pathways, RSV has been extensively studied for its potential to prevent a wide range of illnesses and has been named a “promiscuous molecule” [11]. RSV has demonstrated antioxidative, anti-inflammatory, anticancer, and antiaging effects [12] and has shown promise in preventing the progression of cardiovascular diseases, cancers, ischemic injuries, and neurodegenerative diseases [13]. Importantly, RSV has the ability to cross the blood–brain barrier (BBB) due to its high lipid solubility and its low molecular weight (i.e., 228 Da), making it a potential treatment strategy for AD [14]. However, free RSV suffers from undesirable pharmacokinetic properties, including poor aqueous solubility, low oral bioavailability (< 1%), and extensive metabolism, which limits its therapeutic efficacy [15–17].

It can be suggested that the entrapment of RSV in the nanoparticulate carriers can overcome these limitations and provide an efficient therapy [18]. Lipid-based nanoparticles such as nanostructured lipid carriers (NLCs), solid lipid nanoparticles (SLNs), liposomes, and nanoemulsions are highly considered as the efficient drug delivery systems for poorly water-soluble compounds [19]. However, SLNs are preferred due to their high physical stability, low toxicity, and excellent biocompatibility as well as their high capacity for drug loading (DL%). Moreover, their lipid matrix leads to the incorporation of lipophilic compounds in the core of the nanoparticles which can consequently result in prolonged and sustained release of the lipophilic therapeutics. Furthermore, since the mobility of the compounds is much slower in solid lipids, therefore, incorporation of lipophilic drugs in SLNs caused more reduction in release rate in comparison with other lipid-based formulations. Besides, their ease of manufacturing, along with high reproducibility in their preparation, makes SLNs good carriers for commercial production in a large scale [20–22]. As a colloidal lipid drug delivery system, SLNs with the size range of 50 to 1000 nm are extensively studied and consequently posed low toxicity, cost-effectiveness, high biocompatibility, and biodegradability features, as well as high DL% capacity [23].

Various studies revealed the application of various SLNs in the treatment of AD. Yasir et al. [24] developed a surface-modified NLC for intranasal delivery of donepezil. Vakilinezhad et al. [25] showed that the administration of nicotinamide-containing SLNs can improve symptoms associated with AD as well as reduction in hyperphosphorylation of tau proteins. Rassu et al. [26] developed SLNs for nose-to-brain delivery of BACE1 siRNA in the treatment of AD. Shivananjegowda et al. [27] demonstrated the higher efficacy in the clearance of amyloid beta (A*β*) upon administration of SLNs containing memantine and tramiprosate. Moreover, the encapsulation of various compounds such as ferulic acid [28], piperine [29], curcumin [30], and quercetin [31] into SLNs was studied in the treatment of AD.

In the current study, the RSV-loaded SLNs (RSV-SLNs) were prepared, characterized, and statistically optimized using the Box–Behnken response surface methodology. The physicochemical properties of nanoparticles were evaluated. Moreover, the neuroprotective effectiveness of RSV-SLNs was studied in AD-induced animal models.

## 2. Materials and Methods

### 2.1. Chemicals

SCO, RSV, glycerol monostearate (GMS), 2-thiobarbituric acid (TBA), and DTNB (5,5-Dithiobis2-nitrobenzoic acid) designated as Ellman's reagent were purchased from Sigma (St. Louis, MO, United States). Soy lecithin and Tween 80 were supplied from Samchun Chemical Co., Ltd (Seoul, Republic of Korea). The appropriate kits for the determination of lipid peroxidase (LPO) and GSH were purchased from Navand Salamat Co. (Tehran, Iran). HPLC grade solvents including methanol and acetic acid were obtained from Merck (Darmstadt, Germany). Dialysis bags with molecular cutoffs of 12,000 Da, sodium hydroxide (NaOH), and potassium dihydrogen phosphate (K_2_HPO_4_) were obtained from Merck (Darmstadt, Germany). Ultrapurified water was provided using the Millipore water purification system. All other chemicals were of pharmaceutical grade and used as received.

### 2.2. Preparation and Optimization of RSV-SLNs

In this study, RSV-SLNs were prepared using emulsification and solvent evaporation methods [32]. The aqueous phase was prepared by dissolving Tween 80 in ultrapurified water (20 mL) at 50°C. The organic phase was prepared by dissolving RSV (20 mg), soy lecithin (50 mg), and various quantities of GMS in methanol (5 mL) and kept stirred at 50°C for 10 min until complete dissolution. The organic phase was then added dropwise into the aqueous phase under homogenization at 12,000 rpm for the appropriate period of time, designated as the emulsification time. The resultant oil-in-water (O/W) emulsion was then processed using a rotary evaporator to remove the remaining methanol. For ascertaining in lack of any methanol residue, the evaporation process was continued until no bubble could be visually detected. In order to solidify the nanoparticles, the samples were then stirred in an ice-cold water bath for a period of 15 min. The colloidal dispersion was then centrifuged at 13,000 rpm for 30 min (at 4°C), and the settled-down nanoparticles were separated for further studies. Due to photodegradation of RSV, all procedures were performed in the absence of light, and all containers were covered with aluminum foil.

### 2.3. Characterization of Nanoparticles

#### 2.3.1. Particle Size, Poly Dispersity Index (PDI), and Zeta Potential

The particle size, PDI, and zeta potential of the prepared SLNs were determined by photon correlation spectroscopy (PCS) and laser Doppler velocimetry (LDV), respectively, using a Malvern zetasizer-nanosizer (Malvern Instruments, United Kingdom). All measurements were performed in triplicate at 25°C.

#### 2.3.2. Entrapment Efficiency (EE%) and DL%

The EE% and DL% of RSV in SLNs were determined using the direct method [33]. Briefly, for degradation of SLN structures, the freshly prepared nanoparticles were suspended in methanol followed by sonication for a period of 5 min. Then after, the samples were filtered through a 45-*μ*m-syringe filter (Millipore; MA, United States), and the amount of RSV in the samples was analyzed using high-performance liquid chromatography (HPLC) instrument.

**Figure 1:**
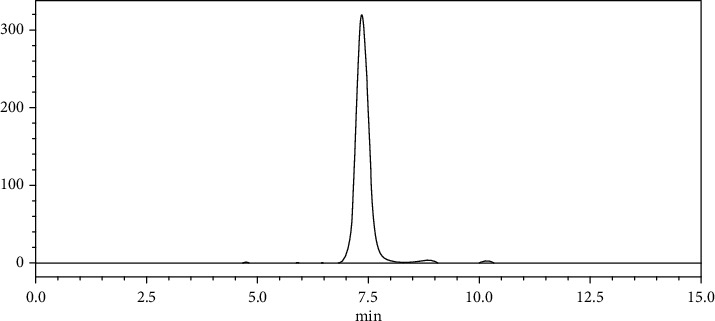
A sample HPLC chromatogram (peak at 7.5 min represents resveratrol).

The HPLC instrument (Shimadzu, Japan) consisted of a pump system (model LC-/20ADXR) and a PDA/SPD-M20A as UV-detector that was set at 303 nm which was the lambda-max of RSV. The lambda-max was found experimentally by scanning the UV absorbance of the compound in the range of 200–400 nm. The HPLC grade water and methanol (40:60 v/v) were used as the isocratic mobile phase. The pH of the mobile phase was adjusted to 3.0 by titration with concentrated acetic acid, and it was delivered at a flow rate of 0.7 mL/min. The stationary phase consisted of a 4.6 × 250 mm C18 column (ODS-3 PerfectSil Target; MZ Company, Germany). The injection value was 20 *μ*L. The related peak for the standard RSV appeared around 7.5 min, and the linear standard curve (*R*^2^ = 0.9981) was depicted in the range of 1–100 *μ*g/mL. A sample HPLC chromatogram of RSV is shown in Figure 1.

After HPLC analysis (*n* = 3), the EE% and DL% were calculated according to the following equations (Equations (1) and Equations (2)):

(1) $$\begin{array}{c} \text{EE}\left(\%\right)=\frac{\text{Amount of Resveratrol in the nanoparticles}}{\text{Total amount of Resveratrol}}\times 100 \end{array}$$

(2) $$\begin{array}{c} \text{DL }\left(\%\right)=\frac{\text{Amount of Resveratrol in the Nanoparticles}}{\text{Total weight of Nanoparticle}}\times 100 \end{array}$$

#### 2.3.3. Experimental Design and Optimization

Pharmaceutical quality by design (QbD) is a systematic approach to pharmaceutical product development for achieving product quality, increasing the capability of the process, and reducing the variability of the product. The design of experiment approach (DoE), as an element of QbD, is recently applied in formulation development [34, 35]. Conventionally, the formulation of drug delivery systems was optimized using univariate techniques designated as one factor at a time in which one variable was changed while others were kept constant. This technique required performing lots of experiments which were cost- and time-consuming. Recently, multivariable techniques such as DoE have been applied alternatively which decreased the number of experiments. Furthermore, in these techniques, the interaction between two variables can be identified, which is not possible in the former approach [36]. In this study and in order to statistically optimize SLNs, the Box–Behnken design (BBD) as one of the elements of DoE was applied [37–39]. The BBD was analyzed using Design-Expert Software (V.7.0.0, Stat-Ease, Inc. Minneapolis, United States) including three independent variables (i.e., factors) as the concentration ratio of GMS/soy lecithin (*A*), the amount of tween 80 (w/v %) (*B*), and the emulsifying time (minute) (*C*). The dependent variables (i.e., responses) were particle size (*Y*_1_) and PDI (*Y*_2_). Initially, EE% was also considered as a dependent factor, but the relevant data was not fitted to an appropriate significant method. Therefore, the response (i.e., EE%) was not considered in the optimization procedure. The ranges of factors as well as the constrains of responses are summarized in Table 1. The independent variables and their ranges were chosen based on a literature review and experimentally performed preliminary studies [40]. Based on the software suggestion, experimental preparations of 15 different batches (F1-F15) were required for mathematical model-fitting and statistical prediction (Table 2).

The significance of the interaction between variables was evaluated using a one-way analysis of variance (ANOVA). The software treated the responses (i.e., particle size (*Y*_1_) and polydispersity index (*Y*_2_)) and determined the best-fitted mathematical model for each response based on several statistical parameters, such as coefficient of variation (CV), R-squared (*R*^2^), adjusted R-squared (adj-*R*^2^), and adequate precision. The level of significance was set at *p* < 0.05. The coefficients of each significant effect were used to obtain a reduced equation through step-wise multiple regression analysis. Furthermore, 3D response surface plots were utilized to provide a visual explanation of certain interactions between the independent variables.

#### 2.3.4. Optimization and Model Validation

After fitting the responses to appropriate mathematical models and model prediction, the software suggested an optimized formulation. In order to model validation using the experimental method, the optimized formulation was experimentally prepared five times, and the related physicochemical properties were determined. The experimentally obtained data were compared to the appropriate data suggested by the software, and the error was calculated.

### 2.4. Freeze Drying

To prepare the lyophilized RSV-SLNs, the sediment cake of the optimized nanoparticles was dispersed in the aqueous solution of mannitol 2% (3 mL). The mannitol was used as the bulking and cryoprotectant agent [40]. To ensure complete reconstitution, the samples were shaken vigorously for a period of 2 min. Then, the samples were transferred to a crystallizer and stored in a freezer at −80°C, overnight. Finally, the samples were dried in a freeze dryer (Operon, South Korea) at −40°C for a period of 72 h to obtain a smooth powder.

### 2.5. Morphological Studies

The morphological evaluation of SLNs was performed using both scanning electron microscopy (SEM) and transmission electron microscopy (TEM). Samples for SEM analysis were prepared by placing some of the lyophilized powder on carbon-coated SEM copper grids covered with a glass lamella. Then, a thin layer of gold was deposited under vacuum using a sputter coater (Moorestown, EUA). The samples were mounted in the SEM chamber, and the imaging of the nanoparticle surface morphology was performed at a voltage of 20 kV.

For TEM, freeze-dried particles were resuspended in freshly prepared distilled water and placed on carbon-coated copper grids. The mixture was dried at room temperature for 5 min, and finally, the size and shape of particles were examined by a CEM 902A (Zeiss, Oberkochen, Germany).

### 2.6. In Vitro Release

A drug release study was done by the dialysis membrane technique [41]. The release test was performed using a shaker incubator (Heidolph Unimax 1010, Germany) at 37°C and agitation rate of 130 rpm. At first, a suitable amount of the lyophilized powder (equivalent to 5 mg of RSV) was redispersed in 5 mL simulated intestinal fluid (SIF) (pH = 6.8), and the colloidal suspension was filled into a dialysis bag which was supplied by Merck (molecular weight cutoff of 12,000 Da). Subsequently, it was immersed into 250 mL of SIF containing Tween 80 (2%) and then stirred for 24 h in the previously mentioned shaker incubator. At the predetermined time intervals, 2 mL of the medium was collected and was replaced with the equal volume of fresh SIF. Considering the aqueous solubility of RSV, the volume of the release medium was chosen in a manner that the establishment of the sink condition was ascertained. Finally, the concentration of RSV in the collected samples was analyzed using the previously mentioned HPLC method.

For a better understanding of the mechanism of in vitro release, the release kinetic study was also performed using curve fitting of all obtained data to various release mathematical models including zero-order, first-order, Higuchi, Korsmeyer–Peppas, and Hixson–Crowell equations. The best-fitted model was selected based on the highest values for correlation coefficient (*R*^2^) and adjusted correlation coefficient (adj-*R*^2^), and the release constant (*K*) was calculated for the best-fitted model.

The mathematical formulation relevant to the Korsmayer–Peppas model is described as follows:

(3) $$\begin{array}{c} \frac{Mt}{M\infty }=K*t^{n} \end{array}$$

where *Mt*/*M*∞ is the fractional amount of drug release, *K* is the kinetic constant, *t* is the time, and *n* is the release component.

In the Korsmayer–Peppas equation, the designated value for the release component (i.e., *n*) can indicate the release mechanism. In cases with *n* < 0.45, the dominant mechanism for release would be the Fickian diffusion. However, an anomalous (non-Fickian) release kinetic, zero-order release kinetics, and super Case-II transport can be considered in 0.4 < *n* < 0.89, *n* = 0.89, and *n* > 0.89, respectively [42].

### 2.7. In Vivo Studies

#### 2.7.1. Animals

Albino Wistar rats, weighing between 180 and 200 g, were purchased from the center of the animal laboratory, affiliated with Hamadan University of Medical Sciences. The animals were kept in the animal house under favorable conditions, including a temperature of 25°C, suitable air humidity (50 ± 5), a 12-h light/dark cycle, and free access to water and food pellets. All animal procedures were registered and approved by the ethical committee of the deputy of research and technology, Hamadan University of Medical Sciences (Registration No.: IR.UMSHA.REC.1399.899).

The animals were randomly allocated into 10 groups (*n* = 6), as indicated in details in Table 3. RSV and RSV-SLNs were suspended in PBS (pH = 7.4) for oral gavage and were administered in the appropriate dosages of 20, 40, and 80 mg/kg/day for 14 consecutive days. For the development of neurodegeneration, SCO was administered to the studied rats by intraperitoneal (IP) injection (2 mg/kg/day) for 14 consecutive days. The dosage of SCO as well as the dosage levels of RSV and the equivalent nanocarrier-based formulations were selected according to previous studies. The Morris water maze (MWM) test was conducted 1 day after the last animal treatment. After the behavioral assessment, the animals were anesthetized using IP administration of ketamine (80 mg/kg)/xylazine (20 mg/kg) and then were sacrificed. The brain tissue samples were removed, washed with ice-colded saline, and preserved at −80°C for further neurobiochemical evaluation [43, 44].

#### 2.7.2. MWM Studies

The MWM test is widely believed to be one of the most effective tests for assessing spatial and memory function in rodents [45, 46]. The apparatus comprises a round water tank that has a diameter of 120 cm and a depth of 60 cm. The tank was filled with water at a temperature of 25 ± 2°C and divided into four equal parts, namely, north, south, east, and west, with the help of some external cues. The white nontoxic color was mixed with the water to give it an opaque appearance. A circular platform, which has a flat metallic top with a diameter of 10 cm, was placed 1 cm below the water surface and fixed in the center of one of the four quadrants. The starting positions for each trial and each rat were selected randomly from north, south, east, and west. The MWM test involves undergoing a 3-day training program that includes three consecutive trials per day. The rats were gently placed into the water, facing the edge of the pool, and allowed to explore the maze for 1 min in four consecutive trials with 5 min intervals between each trial to find and stay on the platform. After staying on the platform for 20 s, the rats were returned to their home cage. During the training, the platform remained in a fixed position, while the starting points were chosen randomly. The mean escape latency time (ELT) of the rat to locate the hidden platform was recorded as an index of learning or acquisition. On the fourth day of the survey, the platform was removed from the pool for a 60-s probe trial test. The index of spatial memory was measured by recording the average duration spent in the target quadrant, the number of entries made to the target quadrant, and the latency time for the first entry into the target quadrant [47].

#### 2.7.3. Preparation of the Brain Tissue

All of the rats were anesthetized using IP administration of ketamine (80 mg/kg)/xylazine (20 mg/kg). The entire brain was quickly removed and rinsed with cold saline to eliminate any blood. The brains were weighed, and a 10% tissue homogenate was created using a 0.025 M Tris-HCl buffer with a pH of 7.5. The resulting mixture was centrifuged at 1000 rpm for 10 min, and the final supernatant was removed to evaluate the further neurobiochemical analysis [44].

#### 2.7.4. Measurement of LPO

The brain LPO was measured by determining bioactive aldehydes such as MDA. Briefly, supernatant (0.01 mL) was mixed with 2 mL of TBA reagent and then heated in a water bath for 15 min. Finally, the mixture was cooled down, the maximum adsorption was read at 532 nm, and the results were reported as nmol/mg of protein [44, 48].

#### 2.7.5. Measurement of GSH

The brain GSH levels were characterized based on the reaction between GSH and Ellman's reagent: DTNB, generating a yellow-colored product. Briefly, the supernatant (1 mL) was mixed with 0.5 mL of Ellman's reagent and 3 mL of phosphate buffer (0.2 M and pH 8.0). The absorbance of samples and standards was measured at 412 nm against blank [44, 49].

#### 2.7.6. Histological Studies

After fixing the samples of brain tissue using 10% formalin solution, paraffin tissue blocks were prepared, and 5-*μ*m thick slices were prepared from each sample with the help of a microtome. Tissue sections were stained with hematoxylin and eosin (H&E). The slides were examined and analyzed using an optical microscope (CX41-Olympus) equipped with a digital camera (Olympus, DP25).

### 2.8. Statistical Analysis

The obtained results were reported as mean ± SD, and the data were analyzed using GraphPad Prism V.8 statistical software (La Jolla, CA, United States). One-way ANOVA and Tukey's post hoc test were used to analyze the data. Also, the difference in the level of data at the level of *p* < 0.05 was used as a criterion for the significance of the data difference.

## 3. Results and Discussion

### 3.1. Preparation and Optimization of RSV-SLNs

In this study, the properties of independent variables, including concentration ratio of GMS/soy lecithin, amount of Tween 80 (milligram), and duration of emulsification time (minute), on the dependent variables, including particle size and PDI of the SLNs, were investigated using the Box–Behnken response methodology. The data, obtained from the experimental preparation of the suggested formulations (i.e., F1-F15), are summarized in Table 2.

To determine the best correspondent models with an appropriate correlation coefficient, the data was fitted to mathematical models using the step-wise method. Each fitted model was characterized by statistical parameters, including CV, *R*^2^, adj-*R*^2^, predicted R-squared (predicted *R*^2^), and adequate precision which were calculated by Design-Expert software. The statistical significance of the variables was determined using ANOVA, while the significance level (*p* value) was considered less than 0.05.

In the current study, statistical analysis of variances revealed that the responses including particle size (*Y*_1_) and PDI (*Y*_2_) were best fitted to linear and quadratic models, respectively. The characteristics of the fitted models are summarized in Table 4.

#### 3.1.1. Particle Size of Nanoparticles

As shown in Table 2, the size of SLNs ranged from 109.3 ± 1.36 to 1096.5 ± 25.76 nm. The best significant fitted model for prediction of the particle size was obtained according to the statistical analysis performed based on BBD. As shown in Table 4, the proposed significant linear model (*p* < 0.001) showed suitable characteristics and proper predictability. The analysis of variance for the proposed model revealed that linear coefficients of two independent factors including GMS/Soy lecithin (*A*) and tween 80% (*B*) were significant (*p* < 0.05). Although a complete ANOVA test was performed to determine the significant factors and their binary interactions, the binary interactions between the variables were found nonsignificant (*p* > 0.05), and therefore, the model was reduced to a linear model using the statistical step-wise method. The complete coefficients of the variables on the particle size are shown in the following equation. However, the value for coefficient *C* was found nonsignificant.

(4) $$\begin{array}{c} Y_{1}=-157.96+362.01\left(\text{A}\right)+276.92\;\left(\text{B}\right)+2.14\;\left(\text{C}\right) \end{array}$$

where *Y*_1_ is the size of nanoparticles (nm), *A* is the linear coefficient of GMS/lecithin concentration ratio, *B* is the linear coefficient for the amount of Tween 80 (mg), and *C* is the linear coefficient for emulsification time (min).

**Figure 2:**
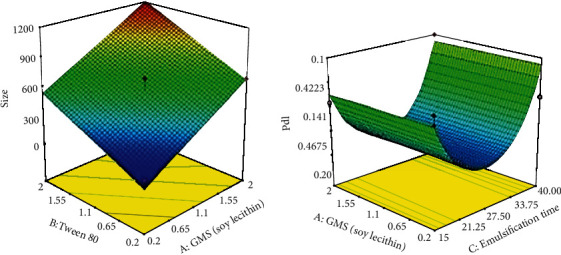
3D response surface plot showing (a) the effect of significant independent variables on particle size and (b) the effect of significant independent variables on PDI.

**Figure 3:**
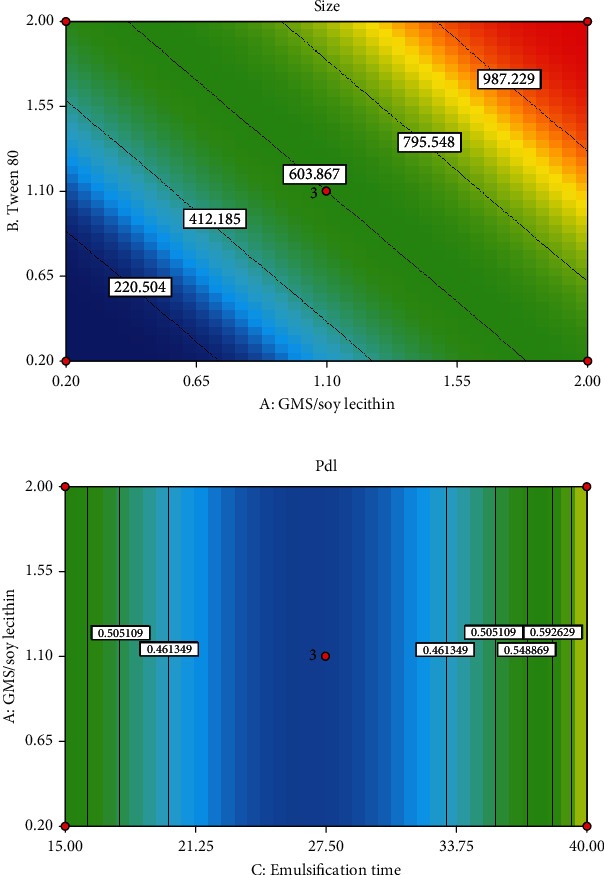
The contour plot showing (a) the effect of significant independent variables on particle size and (b) the effect of significant independent variables on PDI.

As indicated in the equation above, both coefficients of variables *A* and *B* presented a positive effect on the size of the SLNs. Therefore, there is a direct relationship between the size of particles and either the GMS/lecithin concentration ratio (*A*) or the concentration of Tween 80 (*B*). The 3D response surface plot of particle size is shown in Figure 2(a). As depicted in Figure 2(a), the size of nanoparticles was increased by increasing the concentration ratio of GMS/soy lecithin. The same pattern was observed for changing the size of particles due to elevation in the concentration of Tween 80. The contour plot of particle size is shown in Figure 3(a). It is well-known that lipids at high concentrations tend to form coalesce [50, 51].

In this study, GMS, as a lipid matrix and central core of SLNs, has an inevitable role in particle size, and therefore, by increasing the amount of lipid, the size of nanoparticles was raised due to lipid coalescence. Additionally, as the amount of GMS and, therefore, the viscosity of the inner phase increase, the shearing force of the homogenization process would decrease, and this phenomenon can lead to an increase in particle size [52]. Similarly, previous studies demonstrated the increase in the size of SLNs due to an increase in the concentration of lipids [22, 53].

Moreover, according to Figure 2(a), as the Tween 80 concentration increases, the size of the nanoparticles would elevate subsequently. Notably, the elevation in the concentration of surfactant causes an increase in the viscosity of the aqueous phase, consequently leading to poor homogenization efficiency and increasing the size of particles [54]. On the other hand, it has been approved that due to aggregation of the surfactant molecules at the surface of nanoparticles, an increase in the concentration of surfactant can pose an increase in the size of particles. Additionally, some studies suggested that increased concentration of surfactant can lead to excessive intermolecular bonds such as hydrogen bonds between the nanoparticles. This fact can subsequently justify the elevation in size of nanoparticles due to increasing the concentration of surfactant [55, 56].

#### 3.1.2. PDI of Nanoparticles

PDI refers to the uniformity of particle size distribution and homogeneity of nanoparticles, and the value of less than 0.5 is considered as monodispersity of nanoformulation [57]. The PDI values of nanoparticles in this study ranged from 0.3525 ± 0.05 to 0.8195 ± 0.04 (Table 2). The best significant fitted model for prediction of the PDI was obtained according to the statistical analysis performed based on BBD. As shown in Table 4, the quadratic significant model (*p* < 0.001) was best fitted to the data. The analysis of variance for the proposed model revealed that the squared coefficient of one independent factor (i.e., emulsifying time (*C*)) was considered as the only significant factor (*p* < 0.05). The complete coefficients of the variables on the PDI are shown in the following equation. However, the values for coefficients of A, B, A. B, A. C, B. C, A^2^ and B^2^ were nonsignificant.

(5) $$\begin{array}{c} {\text{Y}}_{2}=+1.09+0.16\;\left(\text{A}\right)+0.23\;\left(\text{B}\right)+0.06\;\left(\text{C}\right)–0.09\;\left(\text{A}.\text{B}\right)+0.04\;\left(\text{A}.\text{C}\right)–0.07\;\left(\text{BC}\right)–0.05\;\left({\text{A}}^{2}\right)+0.33\;\left({\text{B}}^{2}\right)–5.17\left({\text{C}}^{2}\right) \end{array}$$

where *Y*_2_ is the PDI of nanoparticles, *A* is the linear coefficient of GMS/lecithin concentration ratio, *B* is the linear coefficient for the amount of Tween 80 (mg), *C* is the linear coefficient for emulsification time (min), A.B is the interaction coefficient between *A* and *B*, BC is the interaction coefficient between *B* and *C*, AC is the interaction coefficient between *A* and *C*, *A*^2^ is the squared coefficient of GMS/lecithin concentration ratio, *B*^2^ is the squared coefficient of for the amount of Tween 80 (mg), *C*^2^ is the squared coefficient of emulsifying time (min).

The 3D response surface plot of PDI is shown in Figure 2(b). As presented in Figure 2(b), by increasing the emulsification time from 15.0 min to 27.5, a sharp decline in PDI values was observed. However, by further elevation of the emulsification time from 27.5 to 40.0 min, a sharp increase was detected in the PDI value. The contour plot for PDI of the particle is shown in Figure 3(b).

#### 3.1.3. Optimization and Model Validation

Table 5 shows the suggested optimized independent factors (i.e., *A*, *B*, and *C*) along with their corresponding predicted responses. To validate the model and calculate appropriate prediction errors using the experimental method, the suggested optimized formulation was prepared and characterized experimentally (*n* = 5).  Table 6 summarizes the observed responses and predicted errors for optimized RSV-SLNs. As shown in Table 6, all prediction errors are below 15% which shows the reliability and predictability of the proposed models.

### 3.2. Morphology of Nanoparticles

**Figure 4:**
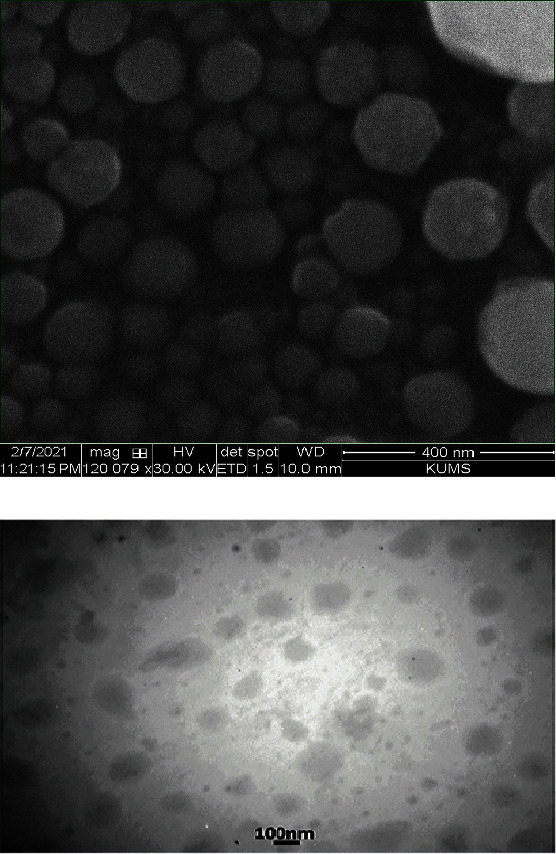
Electron microscopic images: (a) SEM images; (b) TEM images.

The electron microscopic images of the optimized RSV-SLNs are shown in Figures 4(a) and 4(b). The SEM and TEM images indicate that the particles have a smooth surface and spherical shape, and the obtained diameters were in the agreement with those obtained by PCS. As shown in the figure, no evidence of aggregation was observed in the images.

### 3.3. In Vitro Drug Release Study

**Figure 5:**
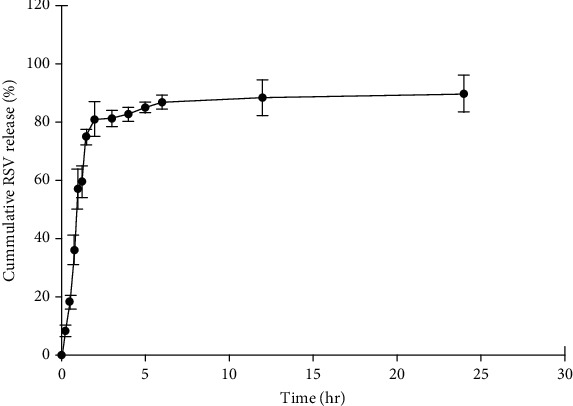
In vitro release profile of RSV from nanoparticles in simulated intestinal fluid (SIF) (*n* = 3).

The investigation of in vitro release of RSV from the optimized SLNs was conducted in SIF, and the pH was adjusted to 6.8. The release profile of the RSV from the nanoparticles is illustrated in Figure 5. As shown in the figure, the release profile exhibited a burst release of approximately 80% of the drug in the first 2 h of postincubation. Afterwards, the profile showed a sustained and controlled release of RSV from nanoparticles in a manner that 93.7 ± 6.54% of the therapeutic compound was released after 24 h postincubation time.

Considering the huge surface area of nanoparticles, the initial burst release is suggested to be attributed to the drug molecules that have been adsorbed to the surface of nanoparticles or precipitated in the superficial lipid matrix [58–61]. Moreover, the sudden release of the active ingredient (AI) from SLNs may be due to the heating and cooling process during homogenization. It was reported previously that the process of heating during homogenization may cause an increase in the solubility of the therapeutic compound in the aqueous phase resulted in the accumulation of the drug in the surface of nanoparticles. Afterwards, the cooling step can lead the oil phase to solidify into a lipid core, which creates a barrier for AI molecules to return to the core [62]. Furthermore, the initial burst release can be justified by the presence of a surfactant agent in the aqueous phase of the dispersion which promotes the solubility of RSV and can lead to the burst release occurrence [63]. The second phase in the in vitro release profile could be associated with the diffusion of AI molecules integrated into the lipid matrix of SLNs. The initial burst release profile could provoke a therapeutic response by supplying a sufficient amount of the drug immediately, and the further sustained release pattern could maintain the therapeutic dose without requiring frequent administration [64]. Similarly, Dara et al. [65] suggested that besides SLNs, other colloidal structures such as mixed micelles could be formed as the result of the interaction of GMS and Tween 80 with the surrounding water. The preparation of these colloidal carriers may be attributed to the initial burst release of the therapeutic compound from SLNs.

The release kinetic study revealed that the obtained data were well-fitted to the Korsmayer–Peppas model (Table 7). In this study, as indicated in the table, *n* (i.e., the release component) is well below 0.0.45 (i.e., 0.1913), suggesting the Fickian diffusion–controlled release of the therapeutic compound from the prepared nanoparticles. Considering that in the SLNs, the drug is mostly incorporated in the lipid matrix; therefore, the solubility of the drug in the lipid structure is the most important factor in defining the release mechanism. RSV, as a lipophilic compound, is highly soluble in the lipid matrix, and therefore, the total amount of the drug is well below the saturation point of RSV. In this case, the release of the drug from the lipid core is mainly by simple diffusion. Some studies suggested diffusion as the prominent mechanism of release from the lipid core, indicating a passive release of the entrapped drug to the release medium [66, 67].

### 3.4. In Vivo Studies

#### 3.4.1. MWM Studies

**Figure 6:**
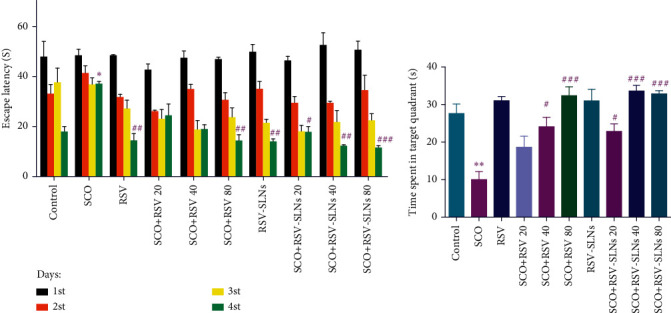
Effect of RSV and RSV-SLN on MWM test in SCO-treated rats. (a) The escape latency time and (b) time spent in the target quadrant. ^∗^*p* < 0.05 and ^∗∗^*p* < 0.01 versus control group; #*p* < 0.05, ##*p* < 0.01, and ###*p* < 0.001 versus SCO group. SCO, scopolamine; RSV, resveratrol; RSV-SLNs, resveratrol-loaded SLNs.

Figure 6(a) represents the results of the escape latency during the consecutive 4 days in various animal groups. It is obvious that the ELT on the fourth day was significantly elevated in the SCO group in comparison to the control group (*p* < 0.01) indicating induction of neurodegeneration by administration of SCO. Moreover, these findings demonstrate that the administration of 80 mg/kg of either RSV or RSV-SLNs in SCO-treated groups can lead to a significant decrease in fourth-day ELT, revealing that the animals were able to find the platform faster compared to the SCO group with *p* < 0.01 and *p* < 0.01, respectively. Interestingly, it was observed that in all SCO-treated animals receiving either 20, 40, or 80 mg/kg RSV-SLN, the fourth-day ELTs were significantly lower than the SCO-treated rats receiving the corresponding doses of free RSV. This finding demonstrates the higher efficacy of RSV-SLN compared with free RSV. In addition, a significant reduction in ELTs was observed in a dose-dependent manner after administration of 20, 40, and 80 mg/kg of either RSV or RSV-SLNs in SCO-treated animals.

For better comparison, the time that each group of animals spent in the target quadrant is shown in Figure 6(b). Similar to the previously mentioned ELTs, the time spent in the target quadrant significantly declined (*p* < 0.01) in SCO-treated rats compared to the control group. However, administration of 40 and 80 mg/kg of RSV could remarkably recover the time spent in the target quadrant in comparison with the SCO-treated rats with *p* < 0.05 and *p* < 0.001, respectively. Additionally, despite the nonsignificant changes in the time spent in the target quadrant between the SCO-treated animals receiving either RSV (80 mg/kg) and SLN-RSV (80 mg/kg), RSV-SLNs could significantly increase the time spent in the target quadrant in 20 mg/kg (*p* < 0.05) and 40 mg/kg (*p* < 0.01) compared to related dosages of free RSV.

The MWM test is widely accepted as one of the most applicable tests to measure spatial memory and learning in rodents. Neurobehavioral anomalies such as impairment of spatial memory and cognitive deficits are specified aspects of neurodegeneration. In this study, it was shown that SCO can induce severe neurobehavioral abnormalities which is in line with previous findings [68–70]. On the other hand, it is demonstrated that although treatment with both free RSV and RSV-SLNs can attenuate spatial memory deficits and improve memory abilities in AD-induced rats, administration of RSV-SLN had more potency for improving the spatial memory rather than administration of free RSV. This was indicated by a reduction in the escape latency and an increase in the time spent in the target quadrant in the RSV-SLN receiving animals.

Similar to this study, in a study performed by Zhang et al. [71], it was demonstrated that IP administration of RSV for a period of 1 month in rats that suffered from vascular dementia can significantly decrease the escape latency and increase the crossing frequency. Moreover, in this study, it was shown that RSV-SLNs in a dose-dependent manner were superior to free RSV in reducing the neuropathological consequence of SCO administration in rats. The observed higher therapeutic potency of RSV-SLNs may be due to improved pharmacokinetic properties of nanoparticles such as increased absorption from GI and decreased hepatic first-pass metabolism. Similar to this study, Ashafaq et al. [72] reported higher efficiency of RSV-loaded NLCs in declining the time to reach the platform in MWM.

#### 3.4.2. Determination of LPO

**Figure 7:**
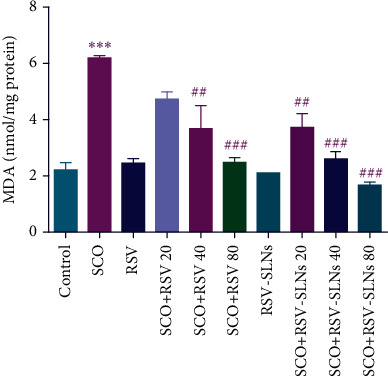
Effect of RSV and RSV-SLN on MDA level in SCO-treated rats. ^∗∗∗^*p* < 0.001 versus control group, ##*p* < 0.05 and ###*p* < 0.001 versus SCO group. SCO, scopolamine; RSV, resveratrol; RSV-SLNs, resveratrol-loaded SLNs.

As shown in Figure 7, SCO-receiving rats showed a significant elevation of MDA levels in the brain samples compared to the control group (*p* < 0.001). However, treatment of rats with free RSV (40 and 80 mg/kg) could significantly decrease the level of the marker with *p* < 0.01 and *p* < 0.001, respectively. Notably, after administration of either 20, 40, and 80 mg/kg of RSV-SLNs in SCO-treated rats, the MDA levels declined in a dose-dependent manner and were significantly lower than the determined values following administration of the related dosages of free RSV with *p* < 0.01, *p* < 0.001, and *p* < 0.001, respectively.

One of the mechanisms by which SCO induces AD-like conditions is oxidative stress [3]. Commonly, the overproduction of reactive oxygen species (ROS) is the harmful outcome of mitochondrial dysfunction after SCO administration. Considering the presence of high amounts of polyunsaturated fatty acids in the brain, this organ can be susceptible to neurodegeneration and neuroinflammation because of LPO. Overall, the level of LPO is assessed based on the formation of active aldehydes like MDA, and its elevation level clearly shows the activity of ROS on the cellular membrane and, therefore, disruption of the integrity and fluidity of the cell membrane due to LPO and oxidative damage [73, 74].

According to the obtained results, following the administration of SCO, the activity of LPO in the brain samples of the studied animals was confirmed by the elevated level of MDA. In addition, the elevated level of MDA indicated that SCO not only impaired spatial memory but also disrupted learning memory, which is demonstrated in this study as well as previous findings [3, 75, 76]. It was previously found that RSV can cause a significant elevation in the expression of antioxidative enzymes such as CAT, GPx, and SOD-1 in the brain tissue [77] and, therefore, can cause a decline in biomarkers of the oxidative stress. In this study, it was shown that treatment with RSV-SLN had the higher potency against SCO neurotoxicity by exertion of a more efficient reduction in the level of MDA in the brain of the studied animals, which could result in the higher efficiency in amelioration of the cognitive and memory deficits. Similarly, the observed results can be endorsed by the findings of Sinha, Chaudhary, and Gupta [78] and Zhou et al. [79].

#### 3.4.3. Determination of GSH

GSH is an essential intracellular antioxidant, is found in all mammalian cells, and plays many important roles including protection against oxidative stress markers such as ROS and reactive nitrogen species (RNS). Abnormalities in mitochondrial activity as well as activating pathological pathways and inflammation are consequences of any alteration in the GSH level in the brain. Due to the fact that high levels of ROS can damage brain tissue, the track of altered GSH concentrations can be observed in many neurological disorders such as ADs [80–82].

**Figure 8:**
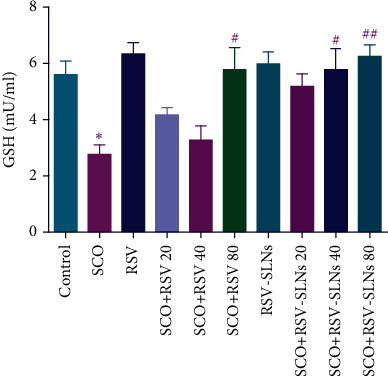
Effect of RSV and RSV-SLNs on GSH level; ^∗^*p* < 0.05 versus control group, #*p* < 0.05 and ##*p* < 0.01 versus SCO group. SCO, scopolamine; RSV, resveratrol; RSV-SLNs, resveratrol-loaded SLNs.

As illustrated in Figure 8, the GSH level in the brain tissue of the SCO-treated group was significantly decreased compared to the control group (*p* < 0.05). However, administration of RSV (80 mg/kg) (*p* < 0.05) and RSV-SLNs (40 and 80 mg/kg) (*p* < 0.05 and *p* < 0.01, respectively) in a dose-dependent manner markedly improved the brain GSH level compared to the SCO-induced AD-like condition.

In normal conditions, free radicals that are produced in the mitochondria can be converted into water and oxygen by the cellular antioxidative system. In the presence of superoxide dismutase (SOD) enzyme, hydrogen peroxide (H_2_O_2_) is generally produced from superoxide anions (O_2_^−^). Under normal conditions, H_2_O_2_ is converted to water and oxygen via catalase (CAT) and glutathione peroxidase (GPx) interference [83]. GSH plays a great role in maintaining cell integrity and minimizing hydrogen peroxide toxicity based on the redox cycle of GSH [84]. However, during stress conditions and due to mitochondrial damage, the production of ROS increases, and the activity levels of antioxidant enzymes decrease. GSH plays a great role in maintaining cell integrity and minimizing hydrogen peroxide toxicity based on the redox cycle [84]. Therefore, the lower activity of GSH can lead to the greater harmful effects of radical species.

In this study, SCO administration was followed by a decrease in GSH levels in the brain tissue, which is in agreement with previous studies [85, 86]. In contrast, it was found that encapsulation of RSV into SLNs led to an increase in the antioxidative activity of free RSV in improving GSH levels. Consequently, higher levels of GSH cause a decrease in oxidative damage and neurobehavioral abnormalities which were induced by SCO. Similarly, previous studies confirm that lipid-based nanoparticles can improve the antioxidative activity of bioactive compounds or medication, exhibiting higher therapeutic potential [72, 87].

#### 3.4.4. Brain Histopathological Alterations

**Figure 9:**
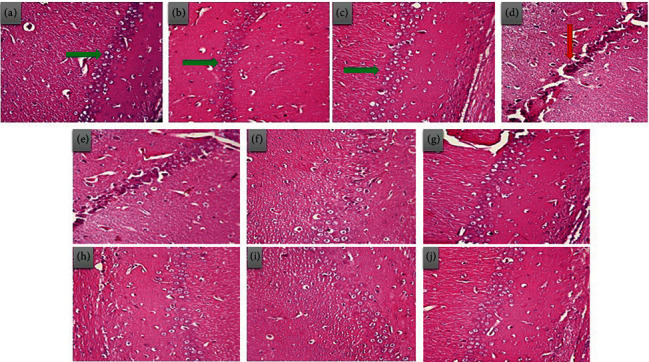
The H&E microscopic examination from the hippocampus CA1 region at 40x magnification. Groups: (a) control, (b) RSV, (c) RSV-SLNs, (d) SCO, (e) Sco+RSV 20 (f) SCO+RSV 40, (g) SCO+RSV 80, (h) SCO+RSV-SLNs 20, (i) SCO+RSV-SLNs 40, (j) SCO+RSV-SLNs 80. SCO, scopolamine; RSV, resveratrol; RSV-SLNs, resveratrol-loaded SLNs. Intact pyramidal cells (green arrow) and dead cells (red arrow).

The H&E section of control, RSV, and RSV-SLNs groups showed the normal histology of the hippocampus cornu ammonis (CA1) region (Figures 9(a), 9(b), and 9(c)). The healthy pyramidal cells are indicated by clear cytoplasm, large vesicular nuclei, and organized cell layer (green arrow). In contrast, SCO administration led to neural degeneration and inflammation, which was indicated by dark, psychotic, and hyperchromatic nuclei, nuclear shrinkage, tangled shape, and disorganized cell layer (Figure 9(d)). Also, congestion and inflammation were observed around the blood capillaries. Notably, administration of 20, 40, and 80 mg/kg of RSV-SLNs, as compared to the RSV treatment group, declined neural degeneration and improved the CA1 pyramidal cell morphology (Figures 9(e), 9(f), 9(g), 9(h), 9(i), and 9(j) ).

Our results revealed that following exposure to SCO, hippocampus CA1 region neurodegeneration occurred, which is in well-agreement with previous studies [88, 89]. It seems that the destruction of pyramidal cells is highly associated with the level of LPO and cognitive and memory impairment due to SCO-induced neural cell injuries. However, RSV-SLN treatment was superior to the RSV treatment to ameliorate hippocampus neural degeneration by inhibition of oxidative injuries and neurodegeneration in male rats. Here, it was shown that RSV encapsulation into SLNs increased the neuroprotective and antioxidative characteristics. Also, due to the more penetration of RSV nanostructures across the BBB, nanodelivery can be considered a promising tool in AD therapy [90, 91].

There are several mechanisms associated with the neuroprotective effects of RSV. Marambaud, Zhao, and Davies [92] reported that although RSV has no significant effect on the production of A*β*, the compound can increase intracellular degradation of the plagues. Moreover, Mishra et al. [93] observed an inhibitory effect of RSV in the elongation and stabilization of A*β* plaques. Marambaud, Zhao, and Davies [92] suggested a decline in the activities of acetylcholine esterase (AchE) and choline acetyltransferase (ChAT) in the cortex and hippocampus of Alzheimer-induced animals that were treated with RSV. Furthermore, they showed that RSV can overexpress Sirt1 in the brains of Alzheimer-induced rats [94]. The neuroprotective effects of Sirt1 are suggested to be through inactivation in transcription of some inflammatory cytokines (i.e., IL1-*β* and IL-6) and activation of NAD-dependent deacetylation of forehead box protein O1 (FOXO) proteins which is highly involved in cellular aging.

Encapsulation of RSV in various nanostructures such as nanocapsules, polymeric micelles, cross-linked chitosan nanoparticles, cobosomes, nanoemulsions, and exosomes for alleviating the AD-associated symptoms are highly studied [95, 96]. Abozaid et al. [97] prepared RSV-selenium nanoparticles and demonstrated higher efficacy of the prepared nanoparticles in the treatment of AD compared with free RSV. Furthermore, Li et al. [98] reported alleviation in symptoms associated with AD using nanocomposites containing RSV and selenium. They showed the efficacy of the nanostructures in the decline in the aggregation of A*β* as well as the exertion of antioxidative properties by decreasing the ROS and enhancing the activation of antioxidative enzymes. The prepared nanostructure also showed higher efficiency in the downregulation of neuroinflammation compared to free components.

## 4. Conclusion

In the current study, the RSV-containing SLNs were prepared for enhancing the oral bioavailability and elaborating the efficacy of RSV in the treatment of AD-associated symptoms. The nanoparticles were prepared, statistically optimized using BBD, and in vitro characterized by the determination of physicochemical properties including particle size, zeta potential, EE%, DL%, and in vitro release. Moreover, in vivo studies showed that the prepared RSV-SLNs had much greater efficiency in the reduction of LPO and increase in GSH levels, as the indicators of antioxidative properties, in the brain of the studied animals. Furthermore, in the MWM study, the ELT as well as the time spent in the target quadrant, as the two indicators of AD-associated symptoms, were significantly improved in animals treated with the prepared nanoparticles. Histopathological studies demonstrated that RSV-containing SLNs exhibited higher potency in the treatment of neural degeneration that occurred in CA1 pyramidal cells. The data obtained in this study revealed that encapsulation of RSV in the SLNs can be considered as the alternative therapy in the amelioration of symptoms related to AD.

However, it should be noted that this study has some constraints. Firstly, the physicochemical stability of the prepared formulation upon storage in certain conditions should be studied. Moreover, further studies are needed to investigate the potential therapeutic efficacy of RSV-SLNs in AD treatment. Moreover, detailed studies should be done on the pharmacokinetic parameters of the prepared RSV-containing SLNs. Furthermore, for translation of these investigations to the patients' beds, the efficacy of the treatment should be investigated in human subjects.

## Figures and Tables

**Table 1:** Ranges and constrains.

**Independent variables (factors)**	**Levels**	
	**−1**	**+1**
A: GMS/soy lecithin ratio	0.2	2.0
B: Tween 80 (w/v %)	0.2	2.0
C: emulsification time (min)	15	40
Dependent variables (responses)	Constrains	
*Y*_1_: size (nm)	Minimize	
*Y*_2_: PDI	Minimize	

**Table 2:** Experimental runs and the response parameters (*n* = 3).

	**Independent variables**			**Dependent variables**	
	**GMS/soy lecithin**	**Tween 80 (w/v %)**	**Emulsifying time (min)**	**Size (nm) (mean ± SD)**	**PDI (mean ± SD)**
**No.**	**(A)**	**(B)**	**(C)**	**(** **Y**_1_ **)**	**(** **Y**_2_ **)**
F1	1.1	1.1	27.5	375.95 ± 4.12	0.4865 ± 0.04
F2	1.1	1.1	27.5	378.4 ± 3.95	0.461 ± 0.03
F3	2	1.1	15	829.45 ± 21.45	0.5555 ± 0.01
F4	1.1	0.2	15	385.8 ± 3.41	0.4635 ± 0.02
F5	2	2	17.5	1096.5 ± 25.76	0.3525 ± 0.05
F6	1.1	0.2	40	328.4 ± 4.55	0.717 ± 0.06
F7	1.1	2	40	1162.4 ± 33.18	0.7515 ± 0.02
F8	0.2	2	27.5	225.15 ± 2.18	0.354 ± 0.04
F9	2	0.2	27.5	687.55 ± 8.64	0.661 ± 0.05
F10	2	1.1	40	1055.75 ± 25.82	0.7 ± 0.03
F11	0.2	0.2	27.5	109.3 ± 1.36	0.3525 ± 0.02
F12	1.1	1.1	27.5	674.2 ± 7.17	0.3996 ± 0.08
F13	0.2	1.1	40	316.2 ± 3.59	0.579 ± 0.06
F14	0.2	1.1	15	412.1 ± 14.37	0.6225 ± 0.07
F15	1.1	2	15	1020.85 ± 21.33	0.8195 ± 0.04

**Table 3:** Animal groups (*n* = 6).

**No.**	**Group name**	**Treatments**
1	Control	Saline solution (ral administration)
2	RSV	RSV (80 mg/kg/day), for 14 consecutive days.
3	RSV-SLNs	RSV-SLNs (80 mg/kg/day), for 14 consecutive days.
4	SCO	SCO (2 mg/kg/day), for 14 consecutive days.
5	SCO + RSV-SLNs 20	SCO (2 mg/kg/day) + RSV (20 mg/kg/day) for 14 consecutive days.
6	SCO + RSV-SLNs 40	SCO (2 mg/kg/day) + RSV (40 mg/kg/day), for 14 consecutive days.
7	SCO + RSV-SLNs 80	SCO (2 mg/kg/day) + RSV (80 mg/kg/day) for 14 consecutive days.
8	SCO + RSV-SLNs 20	SCO (2 mg/kg/day) + RSV-SLNs (20 mg/kg/day) for 14 consecutive days.
9	SCO + RSV-SLNs 40	SCO (2 mg/kg/day) + RSV-SLNs (40 mg/kg/day), for 14 consecutive days.
10	SCO + RSV-SLNs 80	SCO (2 mg/kg/day) + RSV-SLNs (80 mg/kg/day), for 14 consecutive days.

**Table 4:** Characteristics of the best fitted models.

**Dependent variable (responses)**	**Best-fitted model**	**CV (%)**	**R** ^2^	**Adjusted ** **R** ^2^	**Predicted ** **R** ^2^	**Adequate precision**
Particle size (*Y*_1_)	Linear	29.59	0.7784	0.7415	0.6478	14.392
PDI (*Y*_2_)	Quadratic	21.26	0.5492	0.4740	0.3367	4.940

**Table 5:** Optimized independent variable and predicted responses.

**Independent variables (factors)**			**Dependent variables (responses)**	
**GMS/soy lecithin**	**Tween 80 (w/v %)**	**Emulsifying time (min)**	**Size (nm)**	**PDI**
0.23	0.29	26.66	92.77	0.348

**Table 6:** Observed response (*n* = 5).

**Observed responses**						
**Size (nm)**		**PDI**		**Zeta potential (mV)**	**EE (%)**	**DL (%)**
**Observed (mean ± SD)**	**Error (%)**	**Observed (mean ± SD)**	**Error (%)**	**Observed (mean ± SD)**	**Observed (mean ± SD)**	**Observed (mean ± SD)**
104.5 ± 12.3	+11.23	0.322 ± 0.11	−7.47	−3.1 ± 0.15	72.9 ± 5.31	14.6 ± 0.65

**Table 7:** Release kinetic study (*R*^2^: correlation coefficient; adj − *R*^2^: adjusted correlation coefficient; *K*: kinetic constant; *n*: release component).

**Model characteristics**	**Theoretical fitted models**				
	**Zero-order**	**First-order**	**Higuchi**	**Hixson–Crowell**	**Korsmeyer–Peppas**
*R* ^2^	0.2563	0.7476	0.4637	0.0000	0.9648
Adj − *R*^2^	0.2987	0.7281	0.4854	0.0000	0.9589
*K*	—	—	—	—	0.6527
*n*	—	—	—	—	0.1913

## Data Availability

The obtained data was disclosed in the manuscript. The raw data cannot be available due to lab privacy.

## Nomenclature

ACh: acetylcholine
AchE: acetylcholine esterase
AD: Alzheimer's disease
Adj − *R*^2^: adjusted correlation coefficient
AI: active ingredient
ANOVA: one-way analysis of variance
A*β*: amyloid beta
BBB: blood–brain barrier
BBD: Box–Behnken design
CA1: cornu ammonis
CAT: Catalase
Da: Dalton
DL %: drug loading (%)
DSC: differential scanning calorimetry
DTNB: 5,5-dithiobis2-nitrobenzoic acid
EE%: entrapment efficiency (%)
ELT: escape latency time
FOXO: forehead box protein-1
GMS: glyceryl monostearate
GPx: glutathione peroxidase
GSH: reduced glutathione
H&E: hematoxylin and eosin
HPLC: high-performance liquid chromatography
IP: intraperitoneal
K: kinetic constant
LDV: laser-Doppler velocimetry
LPO: lipid peroxidase
MDA: malondialdehyde
MWM: Morris water maze
n: release component
NLC: nanostructured lipid carrier
PCS: photon correlation spectroscopy
PDI: poly dispersity index
QbD: quality-by-design
R^2^: correlation coefficient
ROS: reactive oxygen species
RSV: resveratrol
RSV-SLN: resveratrol-loaded solid lipid nanoparticles
SCO: scopolamine
SEM: scanning electron microscopy
SIF: simulated intestinal fluid
Sirt-1: NAD-dependent deacetylase sirtuin-1
SLN: solid lipid nanoparticles
SOD: super oxide dismutase
SOD-1: superoxide dismutase-1
TBA: 2-thiobarbituric acid
TEM: transmission electron microscopy
